# Facilitation of constructive intra- and inter-personal relationships: A concept analysis

**DOI:** 10.4102/hsag.v29i0.2673

**Published:** 2024-08-20

**Authors:** Andile G. Mokoena-De Beer

**Affiliations:** 1 Department of Nursing Science, School of Health Care Sciences, Sefako Makgatho Health Sciences University, Pretoria, South Africa

**Keywords:** concept analysis, constructive, facilitation, intrapersonal, interpersonal relationships

## Abstract

**Background:**

The researcher’s previous study indicated that couples in a relationship where one partner is diagnosed with borderline personality disorder (BPD) experience intra- and inter-personal difficulties affecting interaction with self and others. Therefore, constructive intra- and inter-personal relationships are essential to facilitate the mental health of couples in a relationship where one partner is diagnosed with BPD. However, the concept has not been defined and applied in caring for such couples.

**Aim:**

The study aims to clarify its meaning by identifying and defining the central concept of ‘facilitation of constructive intra- and inter-personal relationships’.

**Setting:**

The researcher used results from a previous study that explored the experiences of couples in a relationship where one partner is diagnosed with a mental illness to identify and define the central concept.

**Methods:**

The concept was examined using analysis, synthesis, and inductive reasoning strategies, which were applied in two phases.

**Results:**

The central concept of ‘facilitation of constructive intra- and inter-personal relationships’ for couples where one partner is diagnosed with BPD was identified and defined using a dictionary and subject definitions.

**Conclusion:**

Identifying and defining the central concept is essential to developing a model to facilitate constructive intra- and inter-personal relationships.

**Contribution:**

The concept ‘facilitation of constructive intra- and inter-personal relationships’ is unique in its form and valuable for developing a model that can be used as a guiding tool for psychiatric nurses to facilitate the mental health of such couples. Furthermore, the model could benefit other relationships experiencing intra- and inter-personal challenges.

## Introduction

This study presents a concept analysis aimed at informing the formulation of a model for psychiatric nurses to facilitate the mental health of couples in a relationship where one is diagnosed with borderline personality disorder (BPD) (Mokoena 2019). A relationship is the way in which people behave towards one another, whether connected by blood or marriage (Oxford 2008). Relationships are therefore important to create meaningful and positive life experience (Dutton & Ragins 2017). As such sound intra- and inter-personal relationships are essential to create strong bonds in marital relationships. Social bonds and connectedness created through human connections shape the formation of strong bonds that are required to maintain interaction with oneself and others to develop a personality (Siegel 2020). Gardner and Hatch (1989) posit that intrapersonal relationships relate to the value of connectedness, self-awareness, and emotional regulation, while interpersonal relationships are about one’s emotional well-being and strong connection with others to help promote individual growth. In their argument, Mendez-Miller, Naccarato and Radico (2022) and Nuraeni, Komarudin and Mulyana (2021) mentioned that the intrapersonal domain relates to self-management, where one demonstrates control over one’s feelings and mood. Therefore, sound intrapersonal relationships are crucial for personal growth and overall well-being within a relationship.

Howard Gardner and Hatch (1989), the founder of intrapersonal and interpersonal intelligence, postulated that intrapersonal relationships play a role in being able to work and regulate one’s emotions (Gardner 1993; Gardner & Hatch 1989). Both intra- and inter-personal relationships are essential to ensure connectedness. Furthermore, the seminal work of Harry Sullivan (1953) demonstrated interpersonal relationships as essential for interacting with others and developing mental and emotional health (Sullivan 1953). According to Nuraeni et al. (2021), the interpersonal domain relates to how one acts and responds openly to others, which is found in one’s ability to initiate communications, interact and work with others. At the same time, psychological and emotional energies are essential for interacting with others. Both intra- and inter-personal relationships are the foundation of personality development. As such, Sullivan believed that personality is not static as it evolves over time based on the interactions with the environment and the lifespan of individuals, making interactions integral to its development (Jayawickreme et al. 2021).

However, this is different for persons diagnosed with BPD. Persons with BPD are believed to experience chaotic relationships. This is attributed to insecure attachments and the need for them to belong. One of the critical features for those diagnosed with BPD is interpersonal distance, which is linked to a deficit in emotional control (Abdevali et al. 2021). Relationships of persons with BPD are somewhat unstable and experience could be worse for those marital relationships (Jin 2023). Central to this are poor interpersonal relationships and the inability of those with BPD to form strong bonds, which make them appear as difficult (Mokoena 2019). Persons diagnosed with BPD experience considerable anguish and unease because of their fear of being abandoned. This worry can lead to maladaptive behaviours, including impulsiveness, self-harm, and extreme changes in self-perception (Mendez-Miller et al. 2022). Addressing challenges experienced by persons diagnosed with BPD and their relationships with others might help to build healthier patterns of interpersonal relationships and improved coping skills. Individuals with BPD not only pose challenges for their loved ones but also for healthcare professionals, especially nurses, who are key role players in providing care to those diagnosed with BPD leading to negative attitudes towards the individuals (Papathanasiou & Stylianidis 2022). As such, psychiatric nurses could also benefit from interventions that are aimed at improving the mental health of couples in a relationship where one partner is diagnosed with BPD.

It is observed that most studies focused on the experiences of persons with BPD and the relationships of couples where one partner is diagnosed with BPD (Miller, Townsend & Grenyer 2021; Mokoena et al. 2019). However, a few studies focused on interventions that can be used to improve the relationships of such couples (Berry, Davies & Dempster 2017; Bradbury & Bodenmann 2020; Wittenborn et al. 2022). While there was a focus on the role of the nurses in providing care to persons diagnosed with BPD, no study focused on the possible interventions to facilitate the mental health of couples in a relationship where one partner is diagnosed with BPD. Sutherland, Baker and Prince (2020) also noticed the dearth of studies that have led to the development of direct interventions for carers, including nurses, despite the high level of need. Therefore, Miller et al. (2021) recommended that there should be interventions to manage chronic feelings of emptiness experienced by persons diagnosed with BPD extending to their relationship, observing the need to facilitate the mental health of such couples.

There is a dearth of studies in South Africa that focus on the facilitation of couples’ mental health, particularly those in a relationship where one partner is diagnosed with BPD. Against this background, the researcher identified the need to develop a model as a frame of reference for couples in such relationships. To achieve this, the researcher had to conduct a concept analysis by identifying, defining, classifying, and placing the concept into a relationship. Concept analysis seeks to investigate the composition and meaning of a concept. Thus, a concept analysis clarifies the studied concept (Paley 2019). Additionally, concept analysis helps the researcher to refine the concept under study. Consequently, a concept analysis in this study led to the development of a model to facilitate the mental health of couples in a relationship where one partner is diagnosed with BPD.

## Objective

The objective of this article is to provide a comprehensive definition of the central concept of ‘facilitation of constructive intra- and inter-personal relationships’ applicable to the care of couples in a relationship where one partner is diagnosed with BPD.

## Research methods and design

This study followed a qualitative, exploratory, descriptive, contextual method and theory-generating research design guided by a constructivist philosophy of science (Chinn & Kramer 2018; Grove & Gray 2023). Constructivism focuses on understanding the world in which individuals live, including their views of the situation (Creswell & Poth 2025). Thus, constructivist researchers often address the ‘processes’ of interaction among individuals. Constructivism was applicable in this study as the researcher first needed to understand how couples experience being in a relationship where one partner is diagnosed with mental illness and, after that, conduct a concept analysis to define the identified concept. According to Gray and Grove (2021), concept analysis follows a process of defining and naming a previously undefined concept using sources in which the concept is used to establish common elements. It is also used to examine the structure and function of a concept (Walker & Avant 2014).

Therefore, in this study, the concept of ‘facilitation of constructive intra- and inter-personal relationships’ was used as a building block to develop a model as an intervention to facilitate the mental health of couples in a relationship where one partner is diagnosed with BPD. Analysis, synthesis, and inductive reasoning strategies were used to identify and define the central concept. Analysis was used to dissect the concept into components to be better understood, thus bringing clarity (Walker & Avant 2014). Synthesis was used to interpret and attach meaning to the findings of the researcher’s previous study to identify the concept. Additionally, the concept was inductively developed using themes from the interviews describing the couples’ experiences of BPD in their relationship.

Concept analysis was performed in two phases. Phase One identified the central concept of ‘facilitation of constructive intra- and inter-personal relationships’ for couples in a relationship where one partner is diagnosed with BPD from the researcher’s previous study (Mokoena 2019). Phase Two defined the concept and placed it into a relationship, using operational and theoretical definitions from dictionaries and subject-specific sources.

### Ethical considerations

Ethical approval was obtained from the Faculty of Health Sciences Research Committee of the University of Johannesburg, with reference numbers REC-01-17-2017 and HDC-01-18-2017.

## Results

The study was conducted in two phases. In Phase One, the central concepts were identified and in Phase Two, the central concept was defined and classified as described further in the text.

### Phase One: Identification of the central concept

The results from the researcher’s previous study were used to identify the central concept ‘facilitation of constructive intra- and inter-personal relationships’. The researcher’s previous study focused on the experiences of couples in a relationship where one is diagnosed with mental illness. The four themes that led to the identification of the central theme were (1) changed social roles, (2) emotional upheaval, (3) interpersonal distance, and (4) changed relationship with the self (Mokoena et al. 2019). Table 1 provides an overview of the themes and direct quotations from the previous study and is further detailed in Phase Two, which is discussed further in the text.

**TABLE 1:** Themes and direct quotations.

Theme	Direct quotations
Changed social roles	‘We are married, and she cannot work because of her illness’. (Participant 2) ‘Knowing that I am a man who cannot provide for the family’. (Participant 1) ‘But now I feel less of a man because she provides for me. It is difficult to think of my role as a man in this relationship’. (Participant 5)
Emotional upheaval experienced by the individual partners	‘Frustration like it will make me snap’. (Participant 4) ‘It is unnecessary stress for my husband to deal with’. (Participant 9) ‘It hurts me so bad’. (Participant 3)
Interpersonal distance in the couple’s relationship	‘We drift further and further apart’. (Participant 9) ‘There is a lack of communication between us’. (Participant 2)
Changed relationship with the self	‘I feel less of a man because she provides for me’. (Participant 7)

*Source*: Mokoena, A.G., 2019, *A model for psychiatric nurses to facilitate the mental health of couples in a relationship where one is living with borderline personality disorder*, University of Johannesburg, South Africa; Mokoena, A.G., Temane, A., Poggenpoel, M., Myburgh, C.P. & Ntshingila, N., 2022, ‘A model for psychiatric nurses to facilitate the mental health of couples living with borderline personality disorder’, *Gender and Behaviour* 20(3), 20288–20304

The description supplied by the couples indicates that being in such relationships is challenging for both partners. These issues impact how each person relates to themselves and others. Table 1 identified the central concept of ‘facilitation of constructive intra- and inter-personal relationships’. As such, the central, essential, and related concepts originated.

Table 2 provides an overview of central concepts, their classification, and characteristics.

**TABLE 2:** List of central concepts, essential criteria, and related criteria.

Central concept	Essential criteria	Related criteria
Facilitation	Assist Dynamic interactive Process of growth	To make easier Help Improve Create a positive environment Mobilise resources Identify and bridge obstacles Encourage reflection Enhance the quality of life
Constructive	Promote improvement Meaningful learning	Build Useful purpose Produce good results Direct therapeutic process
Intrapersonal relationship	Interacting with self Inner support Achieve goals	Process own thoughts and actions Regulate oneself Understand oneself Individual self and or mind Strive towards growth Increase personal integration
Interpersonal relationship	Relationships with others Communication Social harmony	Deal effectively with others Create meaning Social connection with others Interact with others Engage with people Support healthy lifestyles Live effectively

*Sources*: Mokoena, A.G., 2019, *A model for psychiatric nurses to facilitate the mental health of couples in a relationship where one is living with borderline personality disorder*, University of Johannesburg, South Africa; Mokoena, A.G., Temane, A., Poggenpoel, M., Myburgh, C.P. & Ntshingila, N., 2022, ‘A model for psychiatric nurses to facilitate the mental health of couples living with borderline personality disorder’, *Gender and Behaviour* 20(3), 20288–20304

Consequently, in phase Two, the concept of ‘facilitation of constructive intra- and inter-personal relationships’ was defined as described further in the text.

### Phase Two: Definition of the central concept

The researcher examined central and related concepts to provide a precise conceptual definition, which is the foundation for choosing a suitable operational definition. The necessary characteristics for ‘facilitation of constructive intra- and inter-personal relationships’ were determined. This was accomplished by utilising both dictionary and subject definitions. The dictionary meanings were acquired from both physical and digital dictionaries. The subject definitions were sourced from several academic disciplines, such as psychology, education, psychiatric nursing, and psychiatry. The essential and related criteria of the conceptare identified. The following discussion provides a definition of the fundamental idea that has been selected.

### Dictionary definitions of the concept of ‘facilitation’

Facilitation is *making something easy or easier*, assisting or assisting the progress, or improving something (Vocabulary.com 2024). It can be used as the verb ‘facilitate’, which means to help bring about or *help* something run more smoothly and effectively. Facilitate can be used synonymously with *promote, improve*, or *assist*, freeing from obstruction or difficulty. Merriam-Webster Dictionary (n.d.a.) posits that a facilitator helps to bring an outcome or *helps* individuals to achieve something. Facilitating is an action or *process, helping* people deal with a *process*, especially one that you would like to happen. It also means *making it easier* or ensuring that something happens (Cambridge Dictionary n.d.b.).

### Subject definition of ‘facilitation’

Facilitation in social psychology means improving individual performance in the presence of others. The concept originates from client-centred psychotherapy and theories on helping and helping skills (Bergh & Geldenhuys 2016). As such, a facilitator guides using tools for working effectively. Burrows (1997) analysed the concept of facilitation. They concluded that it is a goal-orientated *dynamic process* in which individuals commit to working together in an environment of genuine mutual respect that enhances critical *reflection*. Facilitation is also used in education, where the teacher, as a facilitator, mobilises *resources* for learning and *encourages* the learners to obtain a comprehensive understanding of self through *reflection* (Hughes & Quinn 2013). In counselling, reflection is a powerful tool for developing a more profound understanding of oneself, thus enhancing the quality of life (Halter 2022; Pretorius, Van Rooyen & Reinbrech-Schutte 2010). A facilitative learning environment is free from obstacles. Therefore, the role of a facilitator in such instances is to identify and bridge obstacles and create a positive environment that encourages *interaction*. Similarly, the Theory for Health Promotion identified facilitation as a *dynamic, interactive* process for promoting health through creating a positive environment and mobilising resources, as well as identifying and bridging obstacles in promoting health (University of Johannesburg 2017). This allows one to engage in a *process of growth*.

### Dictionary definitions of the concept ‘constructive’

According to the Cambridge Dictionary (n.d.a.), constructive means actions that are *useful* to help or *promote* something. Constructive can be used synonymously with the word productive, which means to bring something of. As such, constructing means building or putting something together to produce good results (Cambridge Dictionary n.d.a.). The word ‘construere’ is derived from a Latin word of two parts: ‘con’ and ‘struere’. ‘con’ signifies heaping together and *building* together, and ‘struere’ denotes piling or *building*.

### Subject definition of ‘constructive’

Constructivism is a learning theory based on the work of Piaget, Vygotsky and Bandura, which emphasises *learning* through *building* on existing knowledge (Billings & Halstead 2020). Furthermore, Keating and DeBoor (2018) assert that constructivism helps comprehend social interaction as an attempt to find *meaning*, thus making it an ongoing dynamic learning *process*. In education, the word constructive is used mostly when giving feedback, which must be constructive. Constructive feedback focuses on *producing good results* as it allows for reflection to take place (Bruce & Klopper 2017). According to Chi (2009), positive communication creates *meaningful learning*, which builds mental models and produces output. The concept of ‘constructivism’ is also common in research, which allows for persons to create their own *meaning* from their experiences (Creswell & Poth 2025)

### Dictionary definitions of the concept of ‘intrapersonal relationships’

Intrapersonal intelligence is an adjective that describes the ability to understand and interpret one’s behaviour and emotional state. It can also refer to or reside in a person’s mind (Cambridge Dictionary n.d.d.). Dictionary.com (2024) supports this definition, which further states that intrapersonal means existing or occurring within the self or one’s mind (Merriam-Webster n.d.c.). People with intrapersonal intelligence understand themselves and know their strengths and weaknesses.

### Subject definition of ‘intrapersonal relationships’

Howard Gardner in psychology also played a role in coining the concept of intrapersonal intelligence that involves the art of *self-understanding*. To achieve intrapersonal homeostasis, a person needs to *self-regulate* (Hernani 2021). In psychology, mindfulness programmes are valuable for improving intrapersonal relationships, and their benefits include a greater *understanding of people’s inner* worlds (Altinyelken 2023). The intrapersonal relationships, as defined by the Theory for Health Promotion at the University of Johannesburg (2017), consist of an individual’s internal environment, encompassing the dimensions of the body, mind, and spirit. Intrapersonal relationships encompass the *interactions between people*, families, groups, or communities and their spirituality. Intrapersonal competency is measured when one demonstrates the ability to effectively regulate one’s emotions and behaviour in order to *achieve the goals* one wants (Huerta et al., 2021). Furthermore, intrapersonal competencies can be categorised into two main groups: those that foster *inner support* and resilience and those that enhance the ability to *achieve goals*.

### Dictionary definitions of the concept of ‘interpersonal relationships’

Interpersonal refers to being connected in *relationships with others* or people (Cambridge Dictionary n.d.c.). People with sound interpersonal relationships can form close bonds, communicate, and connect with others. Merriam-Webster Dictionary (n.d.b.) further alludes that interpersonal is an adjective that involves relations between people.

### Subject definition of ‘interpersonal relationships’

Interpersonal is a term studied in social psychology, a subfield of psychology. According to Schacter et al. (2016), interpersonal intelligence was first studied by Howard Gardner (1993), which focused on how one interacts with others. Furthermore, the term is used in interpersonal psychotherapy, focusing on interpersonal skills and social context. Thus, it emphasises self-understanding through *relationships with others* (Schacter et al. 2016). In such therapy, psychologists also offer *support* to build interpersonal skills. In nursing, particularly psychiatric nursing, Hildegard Peplau coined the Theory of Interpersonal Relations, emphasising *communication* skills in interactions with others (Alligood 2018). In such interactions, people form interpersonal systems based on communication and interaction, which later form a social system and can influence personal growth. Communication and respect for one another help prevent conflicts, and in that way, *social harmony* is promoted (Su 2022). According to Yang et al. (2023), social harmony is critical to maintaining positive interpersonal relationships.

### Definition of ‘facilitation of constructive intra- and inter-personal relationships’

Finally, the concept of ‘facilitation of constructive intra- and inter-personal relationships’ is defined as a *dynamic interactive process of growth* that takes place between the psychiatric nurse and the couple in a relationship where one partner is diagnosed with BPD aimed at making the relationship easier for them. During this process, the psychiatric nurse *assists* in creating *meaningful learning* among the couple through interaction with self and providing *inner support* for the couple to *achieve goals* within their relationship. In that way, it promotes improvement in how they interact with themselves and others around them, and ultimately, through better communication, improves their relationships with others and brings social harmony within the couple’s relationship.

## Discussion

The concept of ‘facilitation of constructive intra- and inter-personal relationships’ was defined using dictionary and subject definitions. It was essential to define this concept in the context of psychiatric nursing. This concept is new, and therefore, the role of the researcher was to make it understandable to psychiatric nurses for them to be able to facilitate constructive intra- and inter-personal relationships of couples in a relationship where one partner is diagnosed with BPD (Author in press). The Theory for Health Promotion was essential in defining the concept of ‘facilitation’ from a South African perspective (University of Johannesburg 2017). The constituents of the central concept were used to develop a model for psychiatric nurses to facilitate the mental health of couples in a relationship where one is diagnosed with BPD. The model case used in this study emphasises the significance of psychiatric nurses in the treatment of individuals and couples in a relationship where one partner is diagnosed with BPD and the need to promote their mental health.

### Constructing a model case

This study uses a model case to exemplify the practical application of the concept of ‘facilitation of constructive intra- and inter-personal relationships’, which encompasses all its essential characteristics (Fitzpatrick & McCarthy 2016).

Mr. and Mrs. Luphoko, pseudonyms for a couple, have been in a relationship for over 6 years. They have been acquainted for a decade but have been married for only 6 years. They have a 4-year-old daughter. They are a couple in a relationship where Mrs. Luphoko is diagnosed with BPD while Mr. Luphoko is not. The following is their narrative:

‘I have been in a relationship with Mr. Luphoko for a considerable duration, but our relationship has significantly transformed in the previous 3 years. Prior to receiving a diagnosis of BPD, our relationship was primarily harmonious, although occasional conflicts arose. I was diagnosed with BPD 3 years ago. Following the diagnosis, I started to perceive our connection distinctly. The adjustment encompassed various aspects, including alterations to our roles and household responsibilities.

I encountered difficulties maintaining employment, which hindered my ability to sustain the activities I previously engaged in within the household. Occasionally, I was unable to care for our child. I consistently experienced a sense of being a burden within my household because of the responsibility my spouse had to carry for both myself and our child. I experienced difficulty in managing the circumstances. The conflicts intensified and also affected his family. They began to perceive me distinctly. They made numerous remarks suggesting that I am unworthy of a husband who takes care of both me and our child. They accused me of laziness and a lack of knowledge regarding caring for my family. I consistently held myself accountable for my inability to meet my societal expectations as a female within that household. There was also a notable disparity in our sexual experiences. The situation was highly unfavourable.’ (Mrs. Luphoko, Mother, Person with BPD)

‘I have been present in my wife’s life and our child’s prior to the diagnosis of BPD. Throughout this period, I have consistently provided support and care. However, there are instances where I feel that my efforts may not be sufficient to meet her expectations. Following her diagnosis of BPD, she exhibits a lack of motivation and a diminished desire to engage in work. I have assumed a significant portion, if not the entirety, of the household responsibilities. Occasionally, I would leave for work and return to find her inactive. Consequently, I had to commence by bathing our child, preparing meals, and maintaining cleanliness. I was also obligated to settle payments because of her inability to maintain employment.

Occasionally, I held myself accountable for my lack of support, yet at other times, I believed she had high expectations of me. Consequently, we engaged in persistent conflicts not only with her but also with other members of our family. I no longer derive pleasure from our sexual encounters because of persistent fatigue. I was unable to cope with the demands of the situation. On one occasion, we were fortunate enough to encounter a skilled psychiatric nurse in the ward where my wife was admitted [*Sister Happy*]. The psychiatric nurse offered us assistance and guidance to facilitate the successful functioning of our relationship.

My wife expressed doubt over the service the psychiatric nurse was offering. She was uncertain about its potential benefits and how it would assist our relationship, but she wholeheartedly embraced it. We engaged in a dynamic, interactive process with the nurse to express our frustration. Conversations with the psychiatric nurse facilitated a *process of growth* as we started to exchange information. It became evident to us that engaging with the psychiatric nurse may *promote improvement* during our experience with BPD and foster *meaningful learning* for our interpersonal connection.

The nurse *assisted* us in recognising the necessity of *interacting with ourselves* to provide *inner support* and *achieve goals* in our relationship. We further learnt that interpersonal connections can be achieved through effective *communication* that could benefit our relationships with others and foster *social harmony* within our personal spaces.’ (Mr Luphoko, Father, Person without BPD)

The implementation of structured and professional interventions aimed at fostering constructive intra- and inter-personal relationships for couples experiencing BPD may provide positive outcomes. This intervention has the potential to improve the quality of the couple’s relationship by providing support in navigating the problems associated with partners being diagnosed with BPD.

### Limitations

Concept analysis is not popular in the field of psychiatric nursing, especially concerning couples in a relationship where one partner is diagnosed with BPD. This made it challenging to access literature to review and define the concept of ‘facilitation of constructive intra- and inter-personal relationships’ using scientific literature. However, literature from other subjects was used to define the central concept.

### Recommendations

Recommendations are made for psychiatric nursing practice, education, and research.

#### Recommendations for psychiatric nursing practice

Defining the concept of ‘facilitation of constructive intra- and inter-personal relationships’ is crucial to developing a model that psychiatric nurses can use to support couples affected by BPD. This provides psychiatric nurses with the necessary knowledge to treat couples affected by BPD. Therefore, psychiatric nurses must comprehend the concept to implement it effectively. Psychiatric nurses must be skilled through in-service training and workshops to achieve this. This will enhance knowledge in psychiatric nursing practice and enhance the skills of psychiatric nurses, ultimately improving the care of couples in a relationship where one partner has BPD.

#### Recommendations for nursing education

Teaching such concepts to undergraduate and postgraduate psychiatric nursing students can benefit nursing practice as they will learn about how to manage couples in a relationship where one is diagnosed with BPD. Ultimately, it benefits other relationships where there are persons with any form of mental illness. The couples will benefit from the expertise of psychiatric nurses skilled in facilitating constructive intra- and inter-personal relationships and managing couples where one partner is diagnosed with BPD. Thus, this provides a deeper comprehension of how to deal with such couples. The recommendation is made to curriculum developers to include the facilitation of constructive intra- and inter-personal relationships in nursing to enhance outcomes of individuals, families, and communities diagnosed with mental illness and other health conditions.

#### Recommendations for nursing research

Concept analysis in this study has set the ground for a model to be developed to facilitate the mental health of couples in a relationship where one partner is diagnosed with BPD. This has generated the need for more research to be conducted, which will lead to the implementation and evaluation of the model by psychiatric nurses to facilitate the mental health of couples in a relationship where one partner is diagnosed with BPD. The recommendation is also made for implementing this model in other contexts where individuals and couples are experiencing poor intra- and inter-personal relationships.

## Conclusion

The study elucidates the meaning of the concept of ‘facilitation of constructive intra- and inter-personal relationships’ that is essential to be used as a basis for developing a model for psychiatric nurses to facilitate the mental health of couples in a relationship where one is diagnosed with BPD. The concept is unique and has not been previously defined, adding to the existing psychiatric nursing knowledge. It also emphasises the additional responsibility of psychiatric nurses in supporting the mental health of couples in a relationship when one partner has BPD. It is against these findings that the researcher makes recommendations for nursing practice, nursing education, and nursing research.

## References

[CIT0001] Abdevali, M., Mazaheri, M.A., Besharat M.A., Zabihzadeh, A. & Green, J.D., 2021, ‘Borderline personality disorder and larger comfortable interpersonal distance in close relationships’, *Personality and Individual Differences* 182, 111067. 10.1016/j.paid.2021.111067

[CIT0002] Alligood, M., 2018, *Nursing theorists and their work*, 9th edn., Elsevier, St. Louis, MO.

[CIT0003] Altinyelken, H.K., 2023, ‘The benefits of a mindfulness program for university students: A qualitative exploration on intrapersonal and interpersonal relationships’, *Journal of Humanistic Counseling* 62(1), 25–40. 10.1002/johc.12197

[CIT0004] Bergh, Z. & Geldenhuys, D., 2016, *Psychology in the workplace*, 5th edn., Oxford University Press, Cape Town.

[CIT0005] Berry, E., Davies, M. & Dempster, M., 2017, ‘Exploring the effectiveness of couples interventions for adults living with a chronic physical illness: A systematic review’, *Patient Education and Counseling* 100(7), 1287–1303. 10.1016/j.pec.2017.02.015 28228340

[CIT0006] Billings, D.M. & Halstead, J.A., 2020, *Teaching in nursing: A guide for faculty*, 6th edn., Elsevier Inc, St. Louis, MO.

[CIT0007] Bradbury, T.N. & Bodenmann, G., 2020, ‘Interventions for couples’, *Annual Review of Clinical Psychology* 16, 99–123. 10.1146/annurev-clinpsy-071519-020546 32031866

[CIT0008] Bruce, J. & Klopper, H., 2017, *Teaching and learning the practice of nursing*, 6th edn., Pearson Soth Africa (Pty) Ltd, Cape Town.

[CIT0009] Burrows, D.E., 1997, ‘Facilitation: A concept analysis’, *Journal of Advanced Nursing* 25(2), 396–404. 10.1046/j.1365-2648.1997.1997025396.x 9044016

[CIT0010] Cambridge, n.d.a, ‘Constructive’, in Cambridge Dictionary, viewed 21 March 2024, from https://dictionary.cambridge.org/dictionary/english/constructive.

[CIT0011] Cambridge, n.d.b, ‘Facilitation’, in Cambridge Dictionary, viewed 21 March 2024, from https://dictionary.cambridge.org/dictionary/english/facilitation.

[CIT0012] Cambridge, n.d.c, ‘Interpersonal’, in Cambridge Dictionary, viewed 21 March 2024, from https://dictionary.cambridge.org/dictionary/english/interpersonal.

[CIT0013] Cambridge, n.d.d, ‘Intrapersonal’, in Cambridge Dictionary, viewed 21 March 2024, from https://dictionary.cambridge.org/dictionary/english/intrapersonal.

[CIT0014] Chi, M.T.H., 2009, ‘Active-constructive-interactive: A conceptual framework for differentiating learning activities’, *Topics in Cognitive Science* 1(1), 73–105. 10.1111/j.1756-8765.2008.01005.x 25164801

[CIT0015] Chinn, P. & Kramer, M., 2018, *Integrated theory and knowledge development in nursing*, 10th edn., Elsevier Mosby, St. Louis, MO.

[CIT0016] Creswell, J.W. & Poth, C.N., 2025, *Qualitative inquiry and research design*, 5th edn., Sage, Thousand Oaks, CA.

[CIT0017] Dutton, J.E. & Ragins, B.R., 2017, *Exploring positive relationships at work: Building a theoretical and research foundation*, Psychology Press, New York.

[CIT0018] Fitzpatrick, J. & McCarthy, G., 2016, *Nursing concept analysis: Applications to research and practice*, Springer Publishing Company, LLC, New York, NY.

[CIT0019] Gardner, H., 1993, *Multiple intelligences: The theory in practice*, Basic Books/Hachette Book Group, New York, NY.

[CIT0020] Gardner, H. & Hatch, T., 1989, ‘Multiple intelligences go to school: Educational implications of the theory of multiple intelligences’, *Educational Researcher* 18(8), 4–10. 10.2307/1176460

[CIT0021] Gray, J. & Grove, S., 2021, *Burns and Grove’s the practice of nursing research: Appraisal, synthesis, and generation of evidence practice*, 9th edn., Elsevier, St. Louis, MO.

[CIT0022] Grove, S. & Gray, J., 2023, *Understanding nursing research: Building an evidence-based practice*, 8th edn., Elsevier, St. Louis, MO.

[CIT0023] Halter, M., 2022, *Varcarolis’ foundations of psychiatric-mental health nursing: A clinical approach*, 9th edn., Elsevier, St. Louis, MO.

[CIT0024] Hernani, E.V., 2021, ‘Awareness, groundedness, embodiment: Intrapersonal elements in interpersonal relationships’, in M.P. Levine (ed.), *Interpersonal relationships*, pp. 3–15, IntechOpen, London.

[CIT0025] Huerta, M.V., Carberry, A.R., Pipe, T. & McKenna, A.F., 2021, ‘Inner engineering: Evaluating the utility of mindfulness training to cultivate intrapersonal and interpersonal competencies among first-year engineering students’, *Journal of Engineering Education* 110(3), 636–670. 10.1002/jee.20407

[CIT0026] Hughes, S. & Quinn, F., 2013, *Quinn’s principles and practice of nurse education*, 6th edn., Cengage Learning, London.

[CIT0027] Jayawickreme, E., Fleeson, W., Beck, E.D., Baumert, A. & Adler, J.M., 2021, ‘Personality dynamics’, *Personality Science* 2(1), 1–18. 10.5964/ps.6179

[CIT0028] Jin, J., 2023, ‘Borderline personality disorder’, *Journal of American Medical Association* 329(8), 692. 10.1001/jama.2023.1012 36853248

[CIT0029] Keating, S. & DeBoor, S., 2018, *Curriculum development and evaluation in nursing education*, 4th edn., Springer Publishing Company, LLC, New York, NY.

[CIT0030] Mendez-Miller, M., Naccarato, J. & Radico, J.A., 2022, ‘Borderline personality disorder’, *American Family Physician* 105(2), 156–161. 35166488

[CIT0031] Merriam-Webster, n.d.a, ‘Facilitator’, in *Merriam-Webster.com dictionary*, viewed 21 March 2024, from https://www.merriam-webster.com/dictionary/facilitator.

[CIT0032] Merriam-Webster, n.d.b, ‘Interpersonal’, in *Merriam-Webster.com dictionary*, viewed 21 March 2024, from https://www.merriam-webster.com/dictionary/interpersonal.

[CIT0033] Merriam-Webster, n.d.c, ‘Intrapersonal’, in *Merriam-Webster.com dictionary*, viewed 21 March 2024, from https://www.merriam-webster.com/dictionary/intrapersonal.

[CIT0034] Miller, C.E., Townsend, M.L. & Grenyer, B.F.S., 2021, ‘Understanding chronic feelings of emptiness in borderline personality disorder: A qualitative study’, *Borderline Personality Disorder and Emotion Dysregulation* 8(1), 24. 10.1186/s40479-021-00164-8 34365966 PMC8351135

[CIT0035] Mokoena-de Beer, A.G., 2023, ‘The role of poor interpersonal relationships among couples living with borderline personality disorder’, *Gender and Behaviour* 21(1), 20904–20915.

[CIT0036] Mokoena, A.G., 2019, *A model for psychiatric nurses to facilitate the mental health of couples in a relationship where one is living with borderline personality disorder*, University of Johannesburg, South Africa.

[CIT0037] Mokoena, A.G., Poggenpoel, M., Myburgh, C. & Temane, A., 2019, ‘Lived experiences of couples in a relationship where one partner is diagnosed with a mental illness’, *Curationis* 42(1), a2015. 10.4102/curationis.v42i1.2015 PMC6779990 31588763

[CIT0038] Mokoena, A.G., Temane, A., Poggenpoel, M., Myburgh, C.P. & Ntshingila, N., 2022, ‘A model for psychiatric nurses to facilitate the mental health of couples living with borderline personality disorder’, *Gender and Behaviour* 20(3), 20288–20304. 10.4102/curationis.v44i1.2157 PMC7876966 33567849

[CIT0039] Nuraeni, A., Komarudin, K. & Mulyana, M., 2021, ‘Relationship of intrapersonal and interpersonal intelligence with teamwork and performance of Karate Athletes’, *Competitor* 13(3), 502–517. 10.26858/cjpko.v13i3.24776

[CIT0040] Oxford, 2008, *Oxford popular school dictionary*, Oxford University Press, Cape Town.

[CIT0041] Paley, J., 2019, ‘Reading concept analysis: Why draper has a point’, *Nursing Philosophy* 20(4), 1–10. 10.1111/nup.12252 31136083

[CIT0042] Papathanasiou, C. & Stylianidis, S., 2022, ‘Experiences of futility among nurses providing care to patients with borderline personality disorder in the Greek mental health system’, *Journal of Psychosocial Nursing and Mental Health Services* 60(6), 33–42. 10.3928/02793695-20211119-02 34846225

[CIT0043] Pretorius, D., Van Rooyen, M. & Reinbrech-Schutte, A., 2010, *Patient-centred communication and counselling: Principles and practice*, 1st edn., Juta, Cape Town.

[CIT0044] Schacter, D., Gilbert, D., Wegner, D. & Hood, B., 2016, *Psychology: Second European edition*, Macmillan Education, Palgrave, London.

[CIT0045] Siegel, D.J., 2020, *The developing mind: How relationships and the brain interact to shape who we are*, Guilford Publications, New York.

[CIT0046] Su, F., 2022, ‘Research on the role of interpersonal relationship in school management’, *Journal of Higher Education Research* 3(4), 373. 10.32629/jher.v3i4.913

[CIT0047] Sullivan, H., 1953, *The interpersonal theory of psychiatry*, 1st edn., Routledge, New York, NY.

[CIT0048] Sutherland, R., Baker, J. & Prince, S., 2020, ‘Support, interventions and outcomes for families/carers of people with borderline personality disorder: A systematic review’, *Personality and Mental Health* 14(2), 199–214. 10.1002/pmh.1473 31887229

[CIT0049] University of Johannesburg, 2017, *Department of nursing science paradigm: Theory for health promotion in nursing*, Department of Health Sciences, Johannesburg.

[CIT0050] Walker, L.O. & Avant, K.C. 2014, *Strategies for theory construction in nursing*, 5th edn., Pearson, Harlow, Essex.

[CIT0051] Wittenborn, A.K., Woods, S.B., Priest, J.B., Morgan, P.C., Tseng, C.-F., Huerta, P. et al., 2022, ‘Couple and family interventions for depressive and bipolar disorders: Evidence base update (2010–2019)’, *Journal of Marital and Family Therapy* 48(1), 129–153. 10.1111/jmft.12569 34750834

[CIT0052] Yang, Y., Zhao, M., Dong, Y. & Xia, L., 2023, ‘Longitudinal associations between interpersonal distrust and social aggression during college: Disentangling the within-person process from stable between-person differences’, *Journal of Youth and Adolescence* 53, 1–14. 10.1007/s10964-023-01874-8 37904057

